# Stingray venom activates IL-33 producing cardiomyocytes, but not mast cell, to promote acute neutrophil-mediated injury

**DOI:** 10.1038/s41598-017-08395-y

**Published:** 2017-08-11

**Authors:** Janaina Cardoso dos Santos, Lidiane Zito Grund, Carla Simone Seibert, Elineide Eugênio Marques, Anderson Brito Soares, Valerie F. Quesniaux, Bernhard Ryffel, Monica Lopes-Ferreira, Carla Lima

**Affiliations:** 1 Federal University of Tocantins, Porto Nacional, Brazil; 2 Immunoregulation Unit of the Special Laboratory of Applied Toxinology(CEPID/FAPESP), Butantan Institute, São Paulo, Brazil; 3 Allergy and Lung Inflammation Unit of the Molecular and Experimental Immunology and Neurogenetics (CNRS), Orléans, France

## Abstract

One of the hallmarks of acute inflammation is neutrophil infiltration of tissues. We investigated molecular mechanisms implicated in acute neutrophilic inflammation induced by the venom of a freshwater stingray (*Potamotrygon cf. henlei*) in mice. Ray venom induced early mobilization of neutrophil in the microvasculature of cremaster mice and infiltration of the peritoneal cavity 2 hours after injury, in a dose-response manner. IL-1β, IL-6, TNF-α, and KC were produced. The neutrophilic infiltration did not occur in mice with ST2 receptor and MyD88 adapters neutralized, or in those with PI3K and p38 MAPK signaling blocked. Drastic reduction of neutrophil infiltration to peritoneal cavities was observed in ST2^−/−^, TLR2/TLR4^−/−^, MyD88^−/−^, TRIF^−/−^ and IL-17A^−/−^ mice, and a partial reduction was observed in IL-18R^−/−^ mice. Mast cell Kit W(sh)/W(sh)-, AHR-, NLRP3-, ICE-, IL-1β-, P2RX7-, CD39-, IL-17RA-, and TBX21 *KO* mice retain the ability to induce neutrophilia in peritoneal cavity after ray venom injection. IL-6 and TNF-α alone were insufficient for promote neutrophilia in the absence of ST2 signaling. Finally, abundant production of IL-33 by cardiomyocytes was observed. These results refine our understanding of the importance of the IL-33/ST2 axis and IL-33-producing cardiomyocytes in the early acute neutrophilia induced by freshwater stingray venoms.

## Introduction

Neutrophils, the most abundant immune cells in the circulation, are key players in acute inflammation. They migrate to the site of infection or injury, where they engulf and inactivate microorganisms. Excessive neutrophil recruitment observed in traumatic injuries, autoimmunity, ischemic injuries, and sterile liver injury^1^ and consequent neutrophil activation results in degranulation and release of their vast arsenal of hydrolytic, oxidative, and pore-forming molecules into the extracellular medium, leading to host tissue injury, whereas neutrophil apoptosis contributes to the resolution of inflammation^2^. One of the key observations in effective drug therapy in animal models of neutrophilic inflammation is the decrease in neutrophil influx that correlates with improvement in disease activity. Therefore, understanding mechanisms of neutrophil recruitment is of major physiological and pathophysiological importance.

Recruitment of neutrophils from the bloodstream to inflamed tissues requires a carefully regulated cascade of binding interactions between adhesion molecules on leukocytes and endothelial cells. Neutrophil trafficking is orchestrated by adhesion molecules, such as β2 integrins, chemokines, and cytokines. Neutrophils express a large number of cell surface receptors for recognition of pathogens and the inflammatory environment. Those include G-protein-coupled chemokine and chemoattractant receptors, Fc-receptors, adhesion receptors such as selectins/selectin ligands and integrins, various cytokine receptors, and innate immune receptors, such as Toll-like receptors (TLRs), C-type lectins, Nod-like receptors, and RIG-like receptors^3^. Findings suggest that in addition to established pathways of selectin or chemokine-mediated integrin activation^4^, signaling by distinct TLRs (especially TLR2, TLR4, and TLR5) can activate integrin-dependent neutrophil adhesion^5^. TLR-dependent rapid β2 integrin activation may be extremely important in leukocyte–endothelial interactions in the context of host defense against pathogens or in sterile inflammatory injury.

Stingrays of the family *Potamotrygonidae* are widespread throughout river systems of South America that drain into the Atlantic Ocean. While some members of other families of rays may complete their entire life cycle in freshwater^6, 7^, the potamotrygonid stingrays are believed to be the only group of elasmobranches to have speciated exclusively in freshwaters^8^. Some potamotrygonids are endemic to the upper reaches of the Paraná River and the Tocantins River and their tributaries, causing frequent humans accidents.

Stingray envenomations usually involve puncture wounds and are characterized by immediate, local, intense pain, soft tissue erythematic edema, and necrosis^9^. Envenomation results from the action of toxins produced by venom cells along the spines and by the mucus covering of the ray. In some cases, subsequent bacterial infection may contribute to sequelae. The use of analgesics such as tramadol hydrochloride, carbamazepine, thiamine, non-steroidal anti-inflammatory agents like ketoprofen, or synthetic opioid like meperidine, concomitantly with antibiotics administration, is ineffective to treat the pain or to prevent the development of necrosis^10^.

While venoms of most stingrays remain completely unstudied, venoms of some South American freshwater stingrays have been partially characterized. They contain phosphatidylcholine 2-acylhydrolase, metalloproteinases, hyaluronidase, serine-proteinases and L-amino acid oxidases, C-type lectin-like proteins, human IgE biding allergens (venom allergy-5, allergen Bom p4), and small peptides (porflan and orpotrin)^11–15^.

We have previously shown that *Potamotrygon cf. henlei* venom has inflammatory effect in Swiss mice^16^. Ray venom induced a nociceptive response in mice in a dose-dependent manner, characterized by neurogenic (0–5 min after venom injection) and inflammatory phases (15–40 min). The inflammatory cytokines, IL-1β and IL-6, and also the neutrophilic chemokine, KC, and the macrophage chemokine, MCP-1, were produced in response to intraplantar ray venom injection. Increased vascular permeability and edema were sustained for 5 h in the footpads of ray venom-injected mice. Finally, the intense neutrophilic influx into footpads injected mice was preceded by an increased number of rolling cells and cells adherent to the vascular endothelium.

However, the mechanisms involved in neutrophil trafficking during acute injury with fish venoms are poorly characterized. For a better understanding of the mechanisms involved in the resulting acute neutrophilic inflammation we used intravital microscopy of the inflamed cremaster muscle in mice upon perfusion with venom of the Bigtooth River Stingray (*Potamotrygon cf. henlei*), and a model of peritonitis in gene sufficient or deficient mice, or IL-33/citrine reporter mice. We report that neutrophil mobilization requires a contribution by TLRs and associated adapter molecules MyD88/TRIF and the IL-33/ST2-axis, while mast cells and inflammasome-dependent IL-1β production are dispensable.

## Results

### Coordinated signals involved in neutrophilic response to Potamotrygon cf. henlei venom

**Figure 1:**
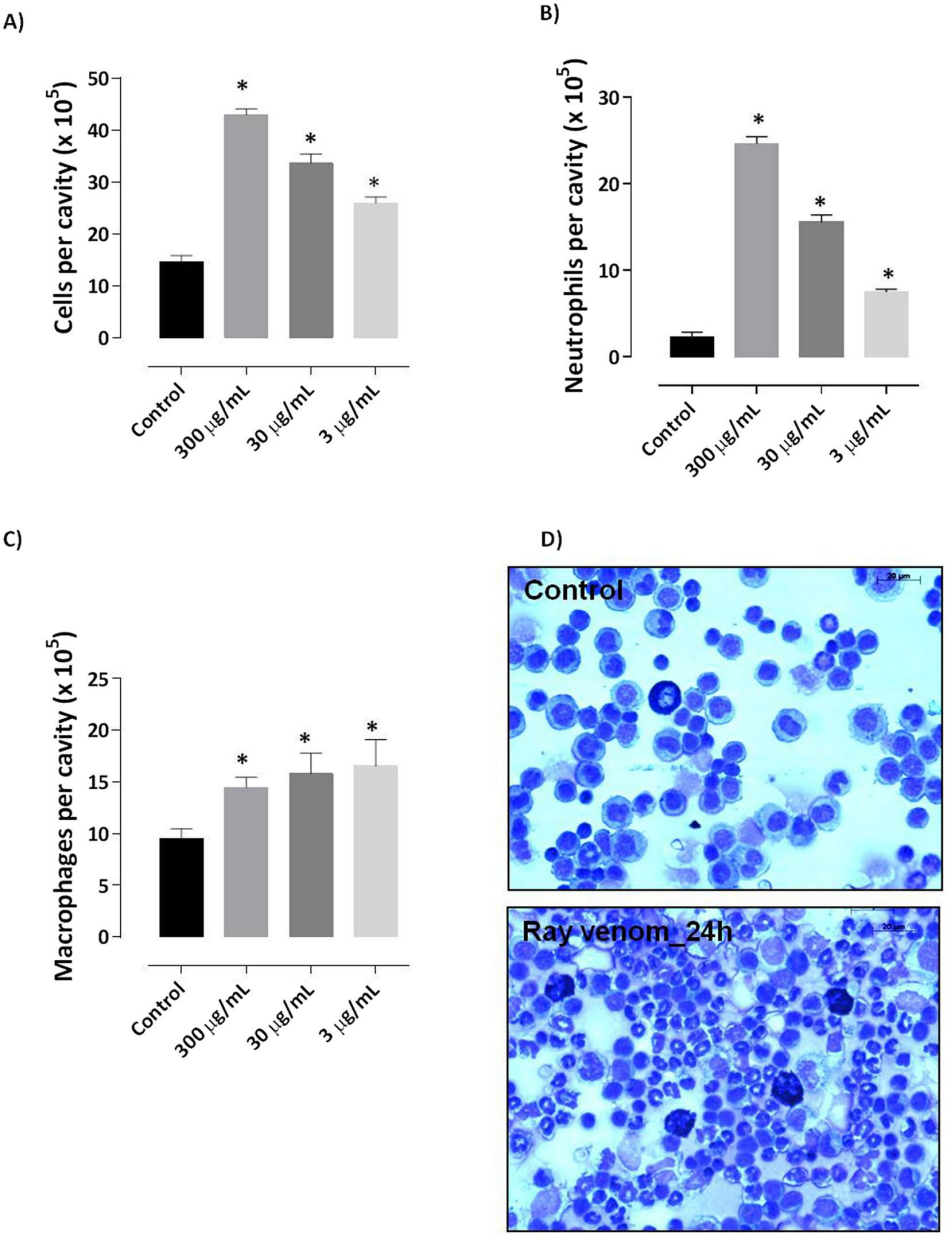
*Potamotrygon cf. henlei* ray venom induces neutrophilia in a murine model of peritonitis. Two hours after i.p. injection of 500 μl of stingray venom at different concentrations (300, 30, or 3 μg/ml), Swiss mice (*n* = 4–7 per group) were killed and peritoneal cavities washed for cell suspension collection. Total (**A**), neutrophils (**B**), and macrophages (**C**) cells were counted. Mice injected with only saline constituted the control-group. Analysis of peritoneal exudates of ray venom-injected mice demonstrate the peak of neutrophils at 24 h (**D**). Results represent mean ± SEM. Pooled results of two independent experiments. *p < 0.05 compared with the control-group.

For the better understanding the mechanism involved in acute neutrophilic inflammation induced by ray venom we first established and characterized a model of peritonitis to analyze the dose-response effect and the kinetic of cell recruitment. Increasing doses (3 to 300 μg/ml) of ray venom induced a significant proportion (30, 46, and 57%) of the peritoneal exudates (Fig. 1A) provoked by neutrophils (Fig. 1B). Changes in the numbers of other immune cells were minimal, with macrophages numbers increasing by 1.6 fold in ray envenomed mice (Fig. 1C). Analysis of peritoneal exudates of ray venom-injected mice demonstrate high neutrophils counts at 24 h compared with control-mice (Fig. 1D).

**Figure 2:**
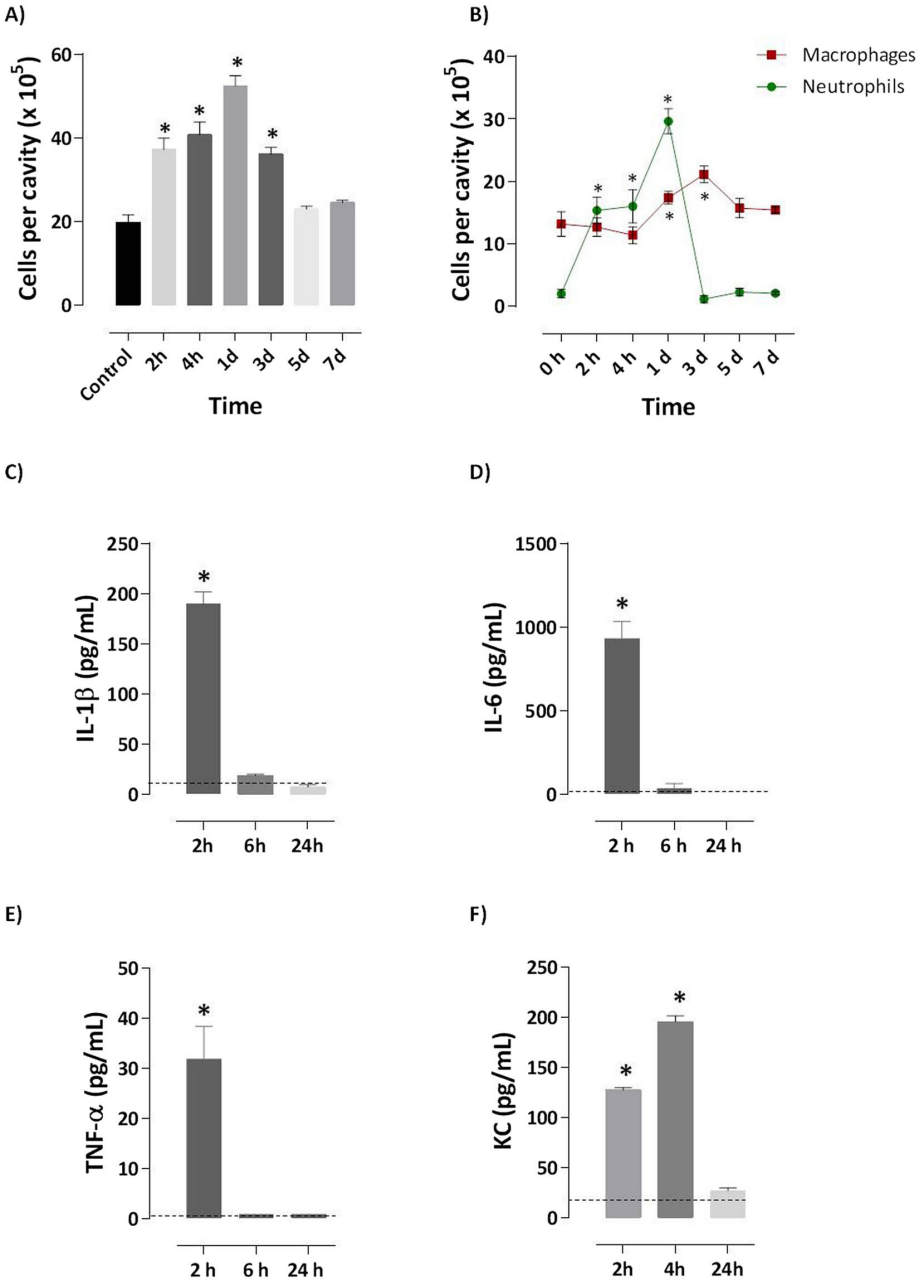
Stingray venom induces neutrophilia with a peak at 24 h after injection. At different time points (2 and 4 h, 1, 3, 5 and 7 days) after i.p. injection of 500 μl of ray venom at 300 μg/ml, Swiss mice (*n* = 4–7 per group) were killed and peritoneal cavities were washed for cell suspension collection. Total (**A**) and neutrophils (**B**) were counted. Levels of IL-1β (**C**), IL-6 (**D**), TNF-α (**E**) and KC (**F**) in exudates of peritoneal cavities of mice were analyzed by ELISA or CBA. Mice injected with only saline constituted the control-group (dotted line). Results represent mean ± SEM. Pooled results of two independent experiments. *p < 0.05 compared with the control-group.

Total leukocyte recruitment increased 2–4 h after ray venom injection (300 μg/ml), reached a peak at 1 day, and remained elevated until day 3, compared to negative control mice (Fig. 2A). Neutrophilia induced by ray venom observed at 2-4 h was sustained until day 1, disappearing after day 3. Macrophages were recruited 1 to 3 days after ray venom injection (Fig. 2B). Analysis of peritoneal exudates from ray venom-injected mice demonstrate that the neutrophils peak at 24 h (Fig. 1D) was preceded at 2 h by production of IL-1β (Fig. 2C), IL-6 (Fig. 2D) and TNF-α (Fig. 2E), and was sustained by the production of KC until 4 h after ray venom injection (Fig. 2F). However, ray venom did not induce the production of IL-2, IL-4, IL-10, IFN-γ, or IL-17A (data not shown).

**Figure 3:**
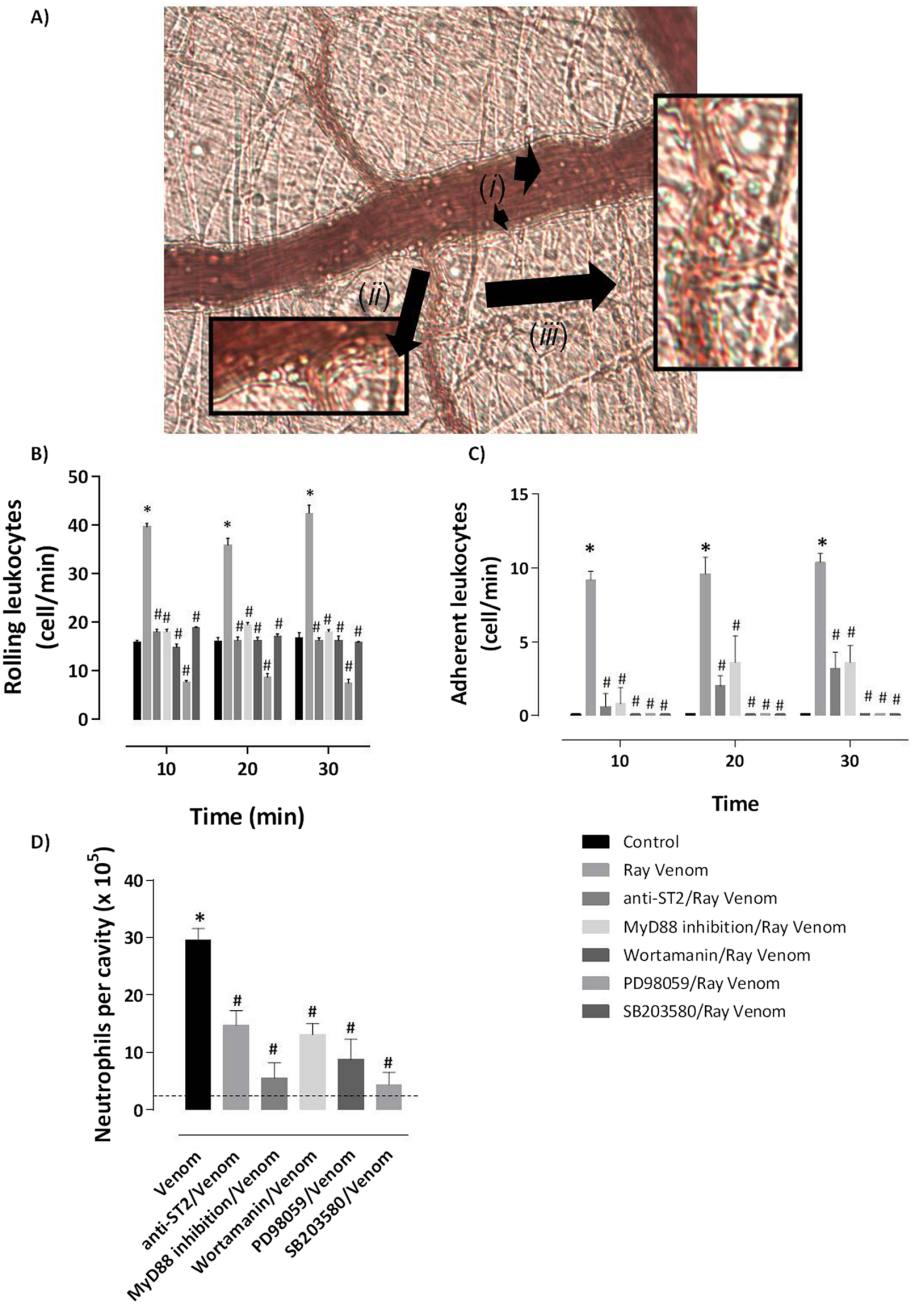
Signals derived from ST2 receptor mobilize leukocytes in post-capillary venules. Swiss mice (*n* = 4–7 per group) were previously treated for 30 min with intraescrotal injection of 5 μg neutralizing anti-ST2 antibody, 100 μM peptide inhibitors of MyD88, 0.005 μM Wortamannin, 2 μM SP98059 or 0.05 μM SB203580. After that, animals were anesthetized and the cremaster muscle were exposed to topical application of 300 μg/ml of stingray venom or sterile saline in 30 µl. Mobilization of leukocytes in post-capillary venules (**A**) was observed for 30 min, and the number of rolling (**B**) and adherent (**C**) leukocytes were counted. The number of neutrophils (**D**) was counted in exudates of peritoneal cavities collected at 2 h from pretreated mice injected with ray venom. Results represent mean ± SEM. Results of two independent experiments are shown. *p < 0.05 compared with the control-group (dotted line) and ^#^ *p* < 0.05 compared to untreated mice that received topically applied ray venom.

Rolling is mostly selectin-dependent, whereas adhesion, crawling, and transmigration depend on integrin interactions. Next, using bright field intravital microscopy we visualize the process of neutrophil recruitment in mouse cremaster muscle in response to ray venom. Intravital microscopy images show captured leukocyte at different stages of migration: freely circulating cells and rolling cells extending tethers (***i***), adhering leukocytes (***ii***) and the cells that had extravasated (***iii***) (Fig. 3A). The leukocytes count in post-capillary venules reveals that neutralization of ST2 receptor interaction by anti-ST2 mAbs and signaling derived from MyD88 adapter by the peptide inhibitor tlrl-pimyd, both inhibited neutrophil rolling (Fig. 3B) and adherence of cells (Fig. 3C) induced by ray venom. Cell rolling and adherent cells were inhibited when phosphatidylinositol (PtdIns) 3-kinases (PI3-kinases or PI3K) was blocked by Wortamannin. Furthermore, inhibition of MEK-1-mediated activation of MAPK by the specific inhibitor PD98059 and of p38 mitogen-activated protein kinase (p38 MAPK) by SB203580 prevented the rolling and adhesion of cells induced by ray venom (Fig. 3B and C).

Next, we evaluated the effect of inhibition of sequential signaling engagement in the acute peritonitis induced by ray venom. Swiss mice that had blocked ST2 receptors and neutralized MyD88 adapters or that had blocked PI3K and p38 MAPK signaling, presented inhibition of leukocyte infiltration (data not shown) into peritoneal cavity, characterized by a drastic inhibition of neutrophils (50–85%) compared to the neutrophilia of ray envenomated group (Fig. 3D).

### Neutrophilic infiltration is mediated by ST2 engagement, but is independent of mast cells or AHR transcription factor

**Figure 4:**
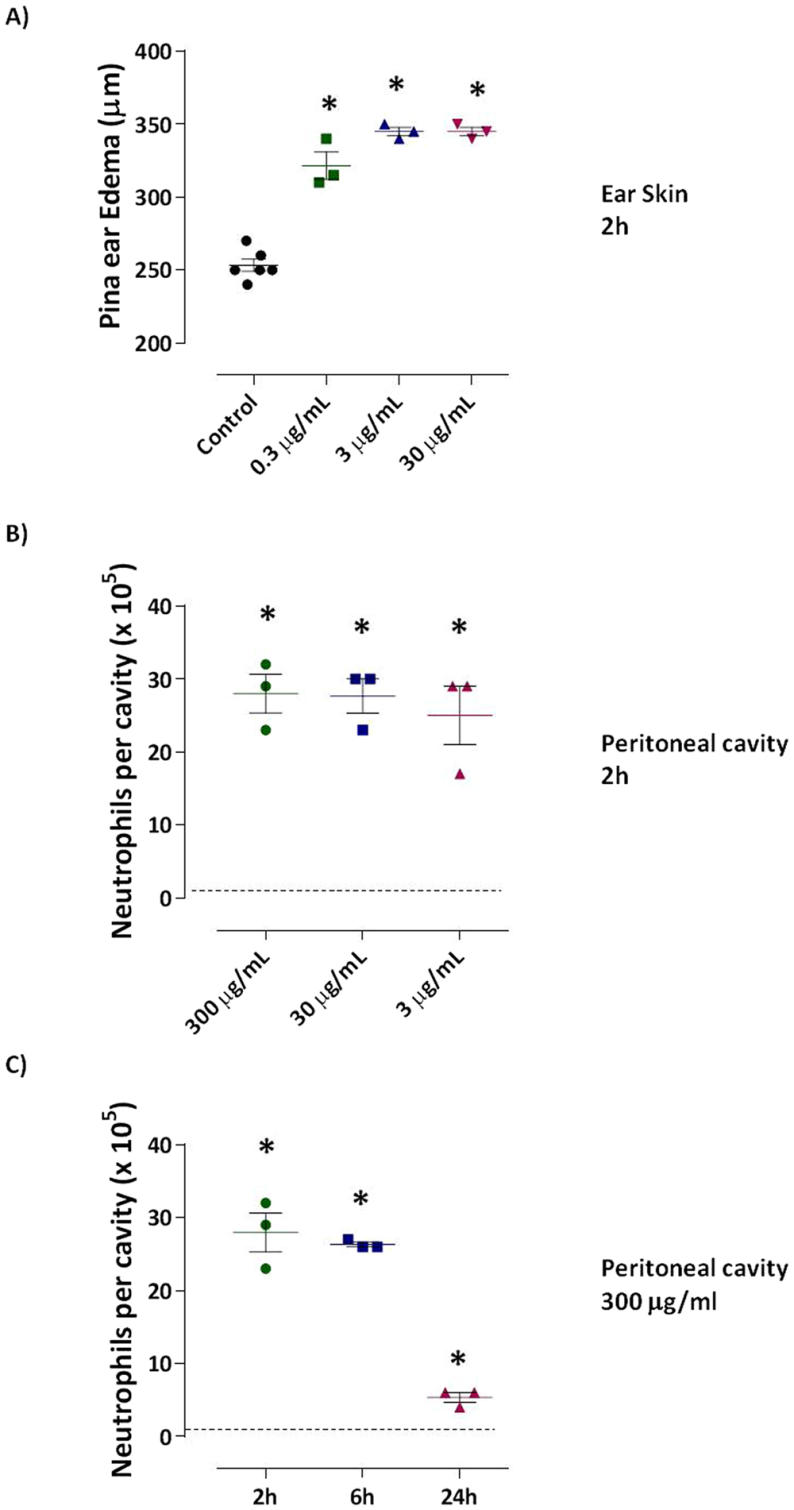
*Potamotrygon cf. henlei* ray venom induces edema formation in C57BL/6. At 2 h after intradermal injection of ray venom at different concentrations (30, 3, or 0.3 μg/ml) into the left ear pinna of C57BL/6 mice (*n* = 3 per group), ear thickness was measured with a microcaliper. The difference in thickness between the left and the right ear was calculated (**A**). The number of neutrophils was counted in peritoneal cavity exudates collected 2 h after mice were injected with different concentrations of ray venom (3, 30, and 300 μg/ml - **B**) or after injection of 300 μg/ml at different time points (2, 6 or 24 h - **C**). Mice injected with only saline comprised the control-group (dotted line). Results represent mean ± SEM. Pooled results of two independent experiments are shown. *p < 0.05 compared with the control-group.

Then, we tested the ability of ray venom to promoted acute inflammation in C57BL/6 *WT* mice by measuring ear swelling. Mice injected intradermally with ray venom (300, 30 or 3 μg/ml) were evaluated after 2 h demonstrating increased swelling at all doses of venom (Fig. 4A). Next, we re-evaluated the dose-response induced by ray venom in this strain of mice. We observed that all doses of ray venom induced recruitment of high and similar number of neutrophils into peritoneal cavity of mice after 2 h (Fig. 4B). Also, the kinetic of cell recruitment induced by ray venom were determined (Fig. 4C). In contrast to Swiss mice, which presented a neutrophils peak at 24 h (Fig. 2B), ray venom at 300 μg/ml promoted high and sustained infiltration of neutrophils until 6 h, followed by a decrease at 24 h in C57BL/6 mice. However, neutrophils were not observed in peritoneal cavities of negative-control mice injected with sterile saline.

**Figure 5:**
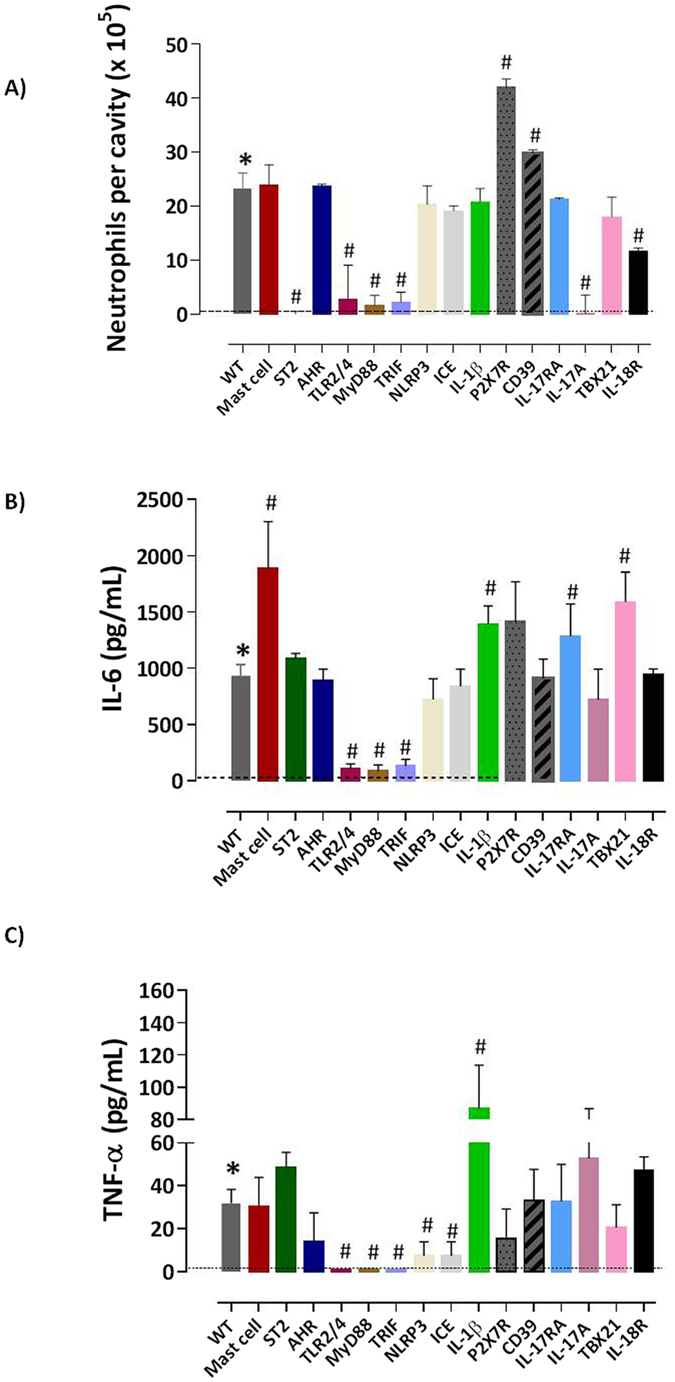
Innate signals regulate the neutrophilia, IL-6 and TNF-α in stingray venom inflammation. Stingray venom (300 μg/ml) in 500 μl or sterile saline were injected i.p. into C57BL/6 *WT* or mast cell-deficient Kit Wsh/Wsh, ST2^−/−^, and AHR^−/−^; or in TLR2/4^−/−^, MyD88^−/−^ and TRIF^−/−^; or in NLRP3^−/−^, ICE^−/−^ and IL-1β^−/−^; or in P2RX7^−/−^ and CD39^−/−^; or in IL-17A^−/−^ and IL-17RA^−/−^, or in TBX21^−/−^ and IL-18R^−/−^. Two hours later, peritoneal cells were harvested and aliquots applied on glass slides were stained and neutrophils (**A**) were counted in a proportion of total cells. IL-6 (**B**) and TNF-α (**C**) were analyzed by CBA in the peritoneal exudates of C57BL/6 *WT* or deficient mice. Results represent mean ± SEM. Pooled results of two independent experiments are shown. *p < 0.05 compared with the control-group (dotted line) and ^#^ *p* < 0.05 compared to *WT* mice injected with ray venom.

IL-33, a member of the IL-1 cytokine family, binds to a heterodimeric receptor comprising ST2 and IL-1 receptor accessory protein (IL-1RAcP), to promote Th2 response and also neutrophilic inflammation^17, 18^. The newly identified ligand-activated transcription factor, aryl hydrocarbon receptor (AHR), resides in the cytoplasm as part of a multiprotein complex and is now known to be involved in various cellular processes, including cell migration and immune functions. Having established that ray venom strongly induces interstitial edema formation (Fig. 4A), we hypothesized the involvement of mast cells activation with attraction of neutrophils to the damaged tissue. To evaluate the role of mast cell-driven neutrophilic inflammation, we challenged C57BL/6 *WT* or Kit Wsh/Wsh *KO* (mast cell-deficient) and ST2 *KO* mice with ray venom. To further explore the role of the AHR as a functionally important molecular target for ray venom, AHR *KO* mice were also used. Our results show that after 2 h, ray venom injection led to an 83% recruitment of neutrophils (comprising 23.3 × 10^5^ cells), whereas in ST2 *KO* mice a drastic reduction in neutrophils recruitment was observed (4%, 0.04 × 10^5^ cells) (Table 1, Fig. 5A). In contrast, the influx of neutrophils into peritoneal cavity of Kit Wsh/Wsh *KO* (65%, 24 × 10^5^ cells) or AHR *KO* (82%, 23.7 × 10^5^ cells) mice was similar to that of C57BL/6 *WT* ray envenomated mice.

**Table 1:** Neutrophilia induced by ray venom is modulated by innate molecules.

Ray Venom (2 h after i.p. injection)	Percentage of Neutrophils in Peritoneal Cavity (%)	Absolute Number of Neutrophils in Peritoneal Cavity (x10^5^)
Negative-control	0	0
**C57BL/6** ***WT***	83.3*	23.3 ± 2.9*
Mast Cell *KO*	64.8	24.0 ± 3.7
**ST2** ***KO***	**4.0** ^**#**^	**0.04 ± 0.5** ^**#**^
AHR *KO*	81.7	23.7 ± 0.4
**TLR2/TLR4** ***KO***	**44.7** ^**#**^	**2.7 ± 6.4** ^**#**^
**MyD88** ***KO***	**42.7** ^**#**^	**1.7 ± 1.8** ^**#**^
**TRIF** ***KO***	**19.5** ^**#**^	**2.3 ± 1.8** ^**#**^
NLRP3 *KO*	63.7	20.4 ± 3.4
ICE *KO*	76.7	19.2 ± 0.9
IL-1β *KO*	71.7	20.8 ± 2.5
P2RX7 *KO*	88.0	42.2 ± 1.4
CD39 *KO*	90.7	29.9 ± 0.6
IL-17RA *KO*	82.0	21.3 ± 0.3
**IL-17A** ***KO***	**14.3** ^**#**^	**0.2 ± 3.4** ^**#**^
TBX21 *KO*	60.3	18.1 ± 3.6
**IL-18R** ***KO***	**48.8** ^**#**^	**11.7 ± 0.6** ^**#**^

### TLR2/TLR4 and MyD88/TRIF signaling mediates neutrophilic infiltration, but independently of inflammasome activation and IL-1β production

Toll-like receptors (TLRs) recognize pathogen associated molecular patterns (PAMPs) in the extracellular milieu, and endosomes induce the priming signals that are necessary for activation of NFκB, the major outcome of TLR signaling. To explore the potential of innate receptors in regulating neutrophilic responses induced by ray venom, TLR2/4, MyD88 or TRIF *KO* mice were used as a model. We observed that the intense recruitment of neutrophils induced by ray venom in peritoneal cavities of *WT* mice (83%, 23.3 × 10^5^ cells) was reduced by 45% in TLR2/4 *KO* mice (2.7 × 10^5^ cells) and in MyD88 *KO* mice (43%, 1.7 × 10^5^ cells) (Table 1, Fig. 5A). Furthermore, a drastic reduction of neutrophil infiltration was also observed in peritoneal cavities of TRIF *KO* mice (19.5% 2.3 × 10^5^ cells).

Nucleotide-binding oligomerization domain (NOD)-like receptor family and pyrin domain containing 3 (NLRP3)-driven activation of caspase-1 are believed to instigate multiple sterile inflammatory disorders^19^. To address the involvement of the critical mediator of inflammation, IL-1β in neutrophilic infiltration induced by ray venom, NLRP3, ICE, and IL-1β were examined (Table 1, Fig. 5A). Infiltration of neutrophils in inflamed tissue was independent of IL-1β/caspase-1/11 activation, since infiltration of neutrophils was not affected by IL-1β (72%, 20.8 × 10^5^ cells), NLRP3 (64%, 20.4 × 10^5^ cells), or caspase-1/11 deficiency (ICE, 77%, 19.2 × 10^5^cells).

One of the endogenous mediators of IL-1β secretion is adenosine triphosphate (ATP), acting via purinergic receptor (P2X) ligand-gated ion channel 7 (P2RX7)^20^. The P1 purinergic receptor, CD39, an ecto-nucleoside triphosphate diphosphohydrolase converts ATP to AMP, thus limiting the concentrations of extracellular ATP. We observed that P2RX7 and CD39 *KO* mice retain the ability to induce neutrophilia in peritoneal cavity (Table 1, Fig. 5A). The absence of P2RX7 increased the infiltration of neutrophils (88%, 42.2 × 10^5^ cells) and the absence of CD39 (91%, 29.9 × 10^5^ cells) did not alter the neutrophilia compared to *WT* ray envenomated mice (83%).

### IL-18R signals improve the neutrophilic inflammation induced by IL-17A

IL-17A-producing cells play protective roles in host defense against certain pathogens, controlling clearance, inflammatory mediator production, and neutrophil recruitment^21^. T-bet is a major transcription factor improving the production of IFN-γ in Th1 cells and inhibiting the IL-17 by Th17. To further explore the role of the IL-17A and IFN-γ as a functionally important cytokines for ray venom inflammation, IL-17A, IL-17RA, TBX21 and IL-18R *KO* mice were also used. We observed a significant reduction in the percentage of neutrophils recruited into peritoneal cavities of IL-17A *KO* (14%, 0.2 × 10^5^ cells), but not in IL-17RA *KO* mice that presented a similar number of neutrophils (82%, 21.3 × 10^5^ cells) after injection of ray venom (Table 1, Fig. 5A). Defects in IL-18R expression in *KO* mice result in partial reduction of neutrophil recruitment (49%, 11.7 × 10^5^ cells), with normal influx of neutrophils (60%, 18.1 × 10^5^ cells) to peritoneal cavities of TBX21 *KO* mice 2 h after ray venom injection.

### Inflammatory cytokines as IL-6 and TNF-α are insufficient to cause neutrophilia in the absence of ST2 signaling

Since the severity of neutrophilia is correlated with the levels of pro-inflammatory cytokines, we compared the levels of cytokines in ray venom injected C57BL/6 *WT* mice with *KO* mice. Our results show that the cytokine IL-6 was induced at 2 h after ray venom injection, and that the mast cell deficiency in Kit Wsh/Wsh *KO* mice promoted an elevation in IL-6 secretion in peritoneal cavity (Fig. 5B). No alterations in the IL-6 production was observed in ST2 or AHR *KO* mice after ray venom injection. In contrast, depletion of TLR2/4, MyD88 or TRIF ablated the production of IL-6 in ray venom-injected *KO* mice. Also, we observe that the deficiency of IL-1β, IL17RA, or TBX21 genes promoted increased IL-6 production in response to ray venom. Similar levels of IL-6 compared to *WT* ray venom-injected mice were observed in NLRP3 and ICE, P2RX7 and CD39, IL-17A, and in IL-18R *KO* mice.

Next, levels of TNF-α were evaluated in *WT* or deficient mice injected with ray venom (Fig. 5C). Normal levels of TNF-α were produced in response to ray venom by mast cell-, ST2- or AHR-deficient mice. In contrast, ray venom did not promote the production of TNF-α in TLR2/4, MyD88, or TRIF *KO* mice compared to *WT* injected mice. Increased levels of TNF-α were induced in IL-1β *KO* mice, but not in NLRP3 and ICE *KO* mice. Similar levels of this cytokine were induced by ray venom in P2RX7 and CD39 *KO* mice, or in IL-17RA and IL-17A *KO* mice, or in TBX21 and IL-18R *KO* mice.

### IL-33-expressing cells is induced by ray venom

**Figure 6:**
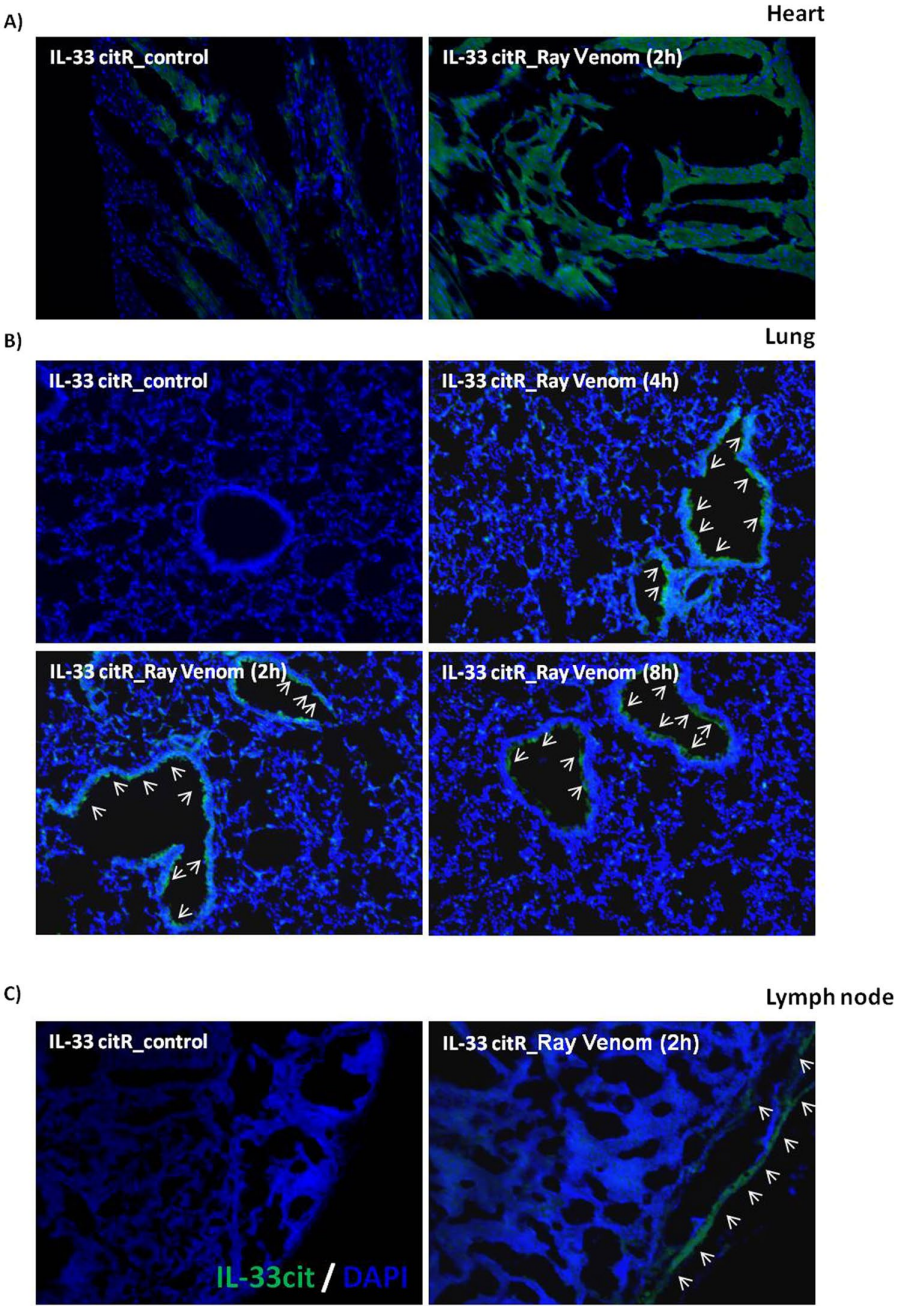
Stingray venom induces IL-33 in heart and lung tissues. Venom (300 μg/ml) in 500 μl or sterile saline were injected i.p. into C57BL/6 *WT* or IL-33/citrine reporter (citR) mice. After 2, 4 or 8 h, mice were anesthetized with a ketamine (50 mg/ml)/xylasine (25 mg/ml) and perfused through the ascending aorta with saline followed by 4% paraformaldehyde. After perfusion, heart (**A**), lung (**B**) and lymph nodes (**C**) were removed and post fixed for two months. For cryosectioning, fixed organs were transferred to 40% sucrose in phosphate buffer, mounted in OCT compound (Neg-50, Richard Allan), sectioned at 15 μm on a cryotome (Cryostat HM 505E), and processed for immunofluorescence. All sections were stained with DAPI. IL-33-produncing cells (indicated by arrows) were imaged with an inverted fluorescence microscope (Olympus BX51 at 10x). Scale bar = 200 μm.

IL-33 resides constitutively in the nucleus, primarily of non-hematopoietic cells, including epithelial cells, endothelial cells, and fibroblasts. Nuclear IL-33 functions as a stored alarmin that is released when barriers are breached^22^. Next, we analyzed whether the neutrophilic inflammation induced by ray venom injury in peritoneal cavity was accompanied by the release of IL-33. Immunohistological analyses of longitudinal sections of heart sections of IL-33/citrine reporter (citR) mice injected with ray venom confirmed abundant production of IL-33 at 2 h in cardiomyocytes. We were also able to detect constitutive low expression of IL-33 in hearts of control-mice (Fig. 6A). The histological examination of lung sections of citR mice receiving i.p. injection of sterile saline (control-mice) revealed a normal cellular architecture and absence of IL-33-expressing cells. However, 2, 4 and 8 h after i.p. injection of ray venom, citR mice presented a significant expression of IL-33 in bronchial epithelial cells (arrows, Fig. 6B). Also, no detection of IL-33 was associated with vascular structures of the heart and lung sections from control- or ray venom-injected citR mice. The sub capsular sinus of lymph nodes is lined by a layer of endothelial cells. Our data revealed that IL-33 was present in the layer of endothelial cells surrounding the sub capsular space of lymph nodes of ray venom citR mice (Fig. 6C). IL-33 production was not observed in control draining lymph nodes. No detection of IL-33-expressing cells was observed in tissues of spleen, liver, or kidney from control- or ray venom-injected IL-33/citrine reporter (citR) mice (data not shown).

## Discussion

The present results demonstrate that the neutrophilic recruitment induced by ray venom depends on coordinated signals generated rapidly in an exudative phase by the PI3K/p38 MAPK pathway, as well as in the late phase after chemoattractant binding of the adapter molecule, MyD88. Our experiments in deficient mice showing that the absence of ST2 severely compromises the acute neutrophilic infiltration, confirm that ST2 engagement is critical in regulating the mobilization of neutrophils to inflamed tissue, independent of its expression on mast cells or the induction of AHR transcription factor. However, our results demonstrate a requirement of TLR/TRIF priming signals for neutrophilia, but demonstrate that participation of NLRP3 inflammasome-derived IL-1β is not required. Our data indicate that in the acute inflammation induced by ray venom, neutrophil recruitment relies on resident cell-derived IL-33, which amplifies innate immunity and cooperates to create an inflammatory microenvironment that triggers accumulation of neutrophils (Supp. Figure 1).

The migration of neutrophils from the circulation to the site of inflammation is controlled by interactions with the vascular endothelium^4^. The prominent pathway triggered by neutrophil GPCRs is the activation of PI3K and subsequent production of PtdIns(3,4,5)P_3_ (PIP_3_) lipid moieties^23^. Our data confirm the requirement of PI3K-dependent mechanism where neutrophil trafficking was directly studied by intravital microscopy of the inflamed cremaster muscle in mice upon perfusion with ray venom. We suggest that activation of G-protein βγ subunits and subsequent activation of p38α MAPK by the chemotactic agent, KC, produced up to 4 h after ray venom injection, could trigger the intrinsic ability of the cells to migrate along those gradient. In neutrophil, p38α MAPK is activated in response to many stimuli, including LPS and TNF-α^24^. Once activated, p38α MAPK is capable of modulating functional responses, including adhesion and synthesis of TNF-α and IL-8 through phosphorylation of transcription factors and activation of other kinases^25^.

Therefore, MAPK signaling pathways downstream of MyD88 appear to be important for neutrophil recruitment in microcirculation of mice triggered by ST2 engagement of IL-33. IL-33 binding by the ST2 receptor leads to the activation and recruitment of MyD88 adapter protein, along with IL-1R-associated kinase1 (IRAK1), IRAK4, and TNFR-associated factor 6 (TRAF6). Events downstream of IL-33/ST2 stimulation may include phosphorylation of extracellular signal-regulated kinase (ERK) 1/2, p38 MAPK, JNKs, and activation of NF-κB^26^.

Mast cells reside in tissues and act as sentinel cells that initiate neutrophil recruitment by controlling and inducing various processes, such as an increase in permeability of local blood vessels and the release of chemokines^27^. Recent studies have provided evidence that mast cells are critical components of innate immune responses that can enhance host defense against the toxicity of arthropod and animal venoms^28^. The aryl hydrocarbon receptor (AHR), a transcription factor known to respond to environmental toxins and endogenous compounds, is present in mast cells. In response to AHR activation, mast cells produce IL-17 and reactive oxygen species, highlighting the potential impact of AHR ligands on inflammation via mast cells^29^.

Recently, using mast cell W(sh)/W(sh) and T1/ST2 deficient mice Enoksson *et al*.^30^ confirmed that IL-33 activates mast cells *in vivo* to recruit neutrophils, a mechanism dependent on IL-33R expression on peritoneal mast cells and on mast cell release of TNF-α. Interestingly, our results show that the ability of ray venom to induce neutrophilia triggered by IL-33/ST2 signals did not require mast cells or AHR. Despite the important ability of mast cells to respond to extrinsic signals, secreting a wide array of inflammatory mediators such as histamine and proteases, our results with mast cell–deficient Kit W(sh)/W(sh) (these mice lack mast cells but have increased levels of basophils)^31^ contrast with those of Kimura *et al*.^32^ that demonstrated a partial role of the mast cell degranulation and histamine release in the formation of edema and the influx of leukocytes in chromolyn-treated *WT* mice injected with *Potamotrygon motoro* venom.

First, our results emphasizes the advantages and limitations of studies of mast cell function using individual mouse models of mast cell deficiency or models that alter expression of mast cell-associated products. On the other hand, this discrepancy reinforces the important role of elements other than mast cells in the control of acute neutrophilia induced by ray venoms.

Moreover, our data highlight the differences between the venoms of terrestrial and aquatic animals. The protein constituents of the venoms and consequently the injuries caused by them differ from those induced by terrestrial venomous animals such as snakes venoms. The composition of South American freshwater stingray venoms have been partially characterized^11–15^. Known constituents include phosphatidylcholine 2-acylhydrolase, metalloproteinases, hyaluronidase, serine-proteinases and L-amino acid oxidases, C-type lectin-like proteins, human IgE biding allergens (venom allergy-5, allergen Bom p4), and two small peptides (porflan and orpotrin). The proteins produced in a typical inflammatory process in post-capillary venules and degraded extracellular matrix components can cause venular stasis, hemorrhage, and changes in the arteriolar wall diameter. These circulatory alterations can explain clinical manifestations observed in human envenomations, as ischemia and necrosis. The infiltration of neutrophils into necrotic zones is associated with exacerbation of the tissue damage.

Palm *et al*.^33^ reported that IL-33 is critical for inducing both Th2 and innate lymphoid cells (ILC) 2 responses to bee venom phospholipase A2 - PLA2. They found that Th2 responses induced by PLA2 were largely dependent on IL-33/ST2 and MyD88 signaling, but were independent of TLR2, TLR4, TLR9, IL-1R, or IL-18R. Also, they found that caspase-1, NLRP3, ASC, and the IL-1 receptor were all required for recruitment of neutrophils to the peritoneal cavity after i.p. injection of bee venom PLA2^19^.

Neutrophils express multiple pattern recognition receptors, including TLR2 and TLR4^34^, which may directly transduce microbe-derived signals. Recently, Zhang *et al*.^35^ demonstrated that signals from the microbes, transduced through TLRs and Myd88, gradually lead neutrophils to become more functionally active. Interestingly, our results demonstrate a requirement of TLR priming signals for the neutrophilia, but rule out the contribution of NLRP3 inflammasome-derived IL-1β. Our data using P2RX7 and CD39-deficient mice confirm that ray venom did not induce accumulation of danger signals, such as ATP and uric acid.

Our data highlight a critical role of IL-1α *in vivo* for neutrophil recruitment. For intraperitoneal ray venom injection, IL-1β/IL-1R signaling or NLRP3_caspase-1/11 activities appeared dispensable for recruitment of neutrophils, showing that another ligand of IL-1R1 receptor as IL-1α can control the cell infiltration at the site of injury. Although IL-1β is inducible and requires posttranslational proteolysis by inflammatory caspases (such as caspase-1) to signal via IL-1R1, IL-1α is constitutively expressed and does not require proteolytic cleavage to become active. IL-1α is also secreted by stressed or damaged cells in a caspase-11-dependent manner in cases of non-canonical inflammasome activation.

IL-1α release following non-canonical inflammasome activation, however, appears independent of NLRP3 or potassium ion efflux. IL-1α functions primarily as a pro-inflammatory cytokine by binding IL-1R1 and activating a MyD88-dependent pathway resulting in NF-κB, JNK and p38 signaling cascades, and in turn activate their own IL-1α and IL-1β production downstream of IL-1R1, chemokine production and recruitment of polymorphonuclear cells to the inflamed tissue^36^.

IL-1β is a highly potent pro-inflammatory mediator at the tissue level that leads to vasodilatation, induces the expression of prostaglandins and promotes the attraction of granulocytes to the inflamed tissue^37^. Therapeutic blockade of IL-1 in humans revealed that some inflammatory diseases are highly dependent on IL-1β, whereas others are less so. Furthermore, our data accord with the view that during the acute phase of inflammation, NLRP3_caspase-1-derived IL-1β has only a minor role in neutrophil recruitment, in contrast to a more dominant role during the chronic phase of active inflammation^38, 39^.

In addition, it is important to note that signals generated by IL-33 binding to the ST2 receptor amplify innate immunity and cooperates to create an inflammatory microenvironment that triggers accumulation of neutrophils. Blockade of IL-33 during MOG-induced EAE ameliorated the disease in part through decreased IL-17 and IFN-γ productions^40^. It is generally thought that IL-17A, the major effector cytokine of the Th17 immune response, induces the expression of neutrophil-attracting chemokines, such as CXCL5^41^.

High amounts of IL-33 are expressed in coronary artery smooth muscle cells and coronary artery endothelium^42^, non-high endothelial venule endothelial cells^43^, cardiac fibroblasts, and cells in an inflammatory milieu^44^, suggesting that IL-33 may play a role in various cardiovascular disorders. Finally, using an IL-33/citrine reporter mice to reproduce the neutrophilic inflammation induced by ray venom, we found that cardiomyocytes are the main cells expressing IL-33, with a smaller, but more prolonged (until 8 h) production by bronchial epithelial cells. However, of IL-33 itself produced by epithelial cells or cardiomyocytes was not enough to drive the recruitment of neutrophils into the lungs or heart of the envenomated mice. IL-33 needs to synergized with other epithelial cytokines and chemokines to induce an inflammatory response. In addition to IL-33 production by sentinel cells, inflammatory cytokines as IL-1α, IL-1β and TNF-α, and mainly CXCR2 receptor binding chemokines as CXCL1 and CXCL2 must be produced locally to direct the transit of neutrophils from the blood into the lungs. Chemokines act locally to induce neutrophils to exit the vasculature into peripheral tissue. In a model of acute lung injury, activation of CXCR2 was not only required on neutrophils, but also on endothelial cells^45^.

The biologic significance of our findings is the essential role of the IL-33/ST2 pathway in the accumulation of neutrophils in response to envenomation induced by *Potamotrygon cf. henlei*. We confirm that the acute neutrophilic infiltration in injured tissue could be associated with the greater perturbation of sentinel cells provoked by ray venom. Components of the ray venom such as collagenolytic proteases can degrade extracellular matrix (ECM) components, inducing endothelial and epithelial cell damage accompanied by the release of IL-33 alarmin. Endothelial cells receive information directly from the actions of other venom toxins and integrate it to generate cellular responses and to augment the activation of remote sentinel cells and epithelial, endothelial, or other immune cells, leading to production of inflammatory cytokines and more IL-33. These mediators fine-tune barrier functions and promote infiltration of neutrophils into necrotic zones, exacerbating tissue damage.

Infiltrating neutrophils are detrimental to vascular integrity and blood flow, promoting tissue damage by releasing reactive oxygen/nitrogen species, myeloperoxidase, serine proteases, elastase, cathepsin-G, MMP-9 from granules, lipid mediators, and TNF-α, IL-1β, IL-17A, and IL-8. However, excessive priming of neutrophil NADPH oxidase results in production of excessive reactive oxygen species that contribute to tissue damage and a heightened inflammatory reaction. Activated neutrophils form neutrophil extracellular traps (NETs) implicated as harmful contributors to various sterile inflammatory conditions^46, 47^. Lefrancais *et al*.^48^ showed that neutrophil serine proteases, cathepsin G, and elastase can generate mature forms of IL-33 that have greatly increased biological activity (∼10 fold) compared to the uncleaved protein. This suggests that proteolytic processing of IL-33 by neutrophils may be required for amplification of inflammatory responses.

To date, no effective drugs targeting the underlying pathophysiology have been proven to improve clinical outcomes of fish envenomation. Our data illustrate the role of IL-33/ST2 in the pathogenesis of stingray envenomation via neutrophil recruitment. Accordingly, this work suggests that a pharmaceutical with anti-IL-33 activity might substantially improve patient outcomes in stingray envenomations by reducing neutrophils infiltration.

## Materials and Methods

### Mice

Male Swiss mice were obtained from a colony at the Butantan Institute. All mice, 6-to 8 week old, were kept under pathogen-free conditions and given water and food *ad libitum*. They were housed under controlled temperature, humidity, and lighting conditions. All procedures involving mice were in accordance with guidelines provided by Brazilian College of Animal Experimentation and approved by the Animal Ethics Committee of Butantan Institute (number 1370/15). Male or female mice, 5–6 weeks old, including C57BL/6 wild type (*WT*) and Mast cell Kit W(sh)/W(sh)-, ST2-, AHR-, IL-1β-, IL-17RA-, IL-17A-, P2RX7-, TBX21-, MyD88-, TRIF-, NLRP3-, ICE-, TLR2/4-, CD39-, and IL-18R-deficient mice – *KO* (all on a C57BL/6 background) and IL-33/citrine reporter (citR) mice were obtained from Transgenose Institute (Orleans, France). All animal experiments complied with the French government’s ethical and animal experiment regulations. Mice were maintained in sterile microisolators with sterile rodent feed and acidified water, and were housed in positive-pressure air-conditioned units (25 °C, 50% relative humidity) on a 12 h light/dark cycle.

### Potamotrygon cf. henlei ray venom preparation

All necessary permits for capture *Potamotrygon cf. henlei* and to collect their venom were obtained from the *Instituto Brasileiro do Meio Ambiente e dos Recursos Naturais Renováveis* - IBAMA (Permit Number: 45 407-1). Stingrays were transported to the laboratory and were anesthetized with 2-phenoxyethanol prior to sacrifice. Adult female and male (*n* = 15) *Potamotrygon cf. henlei* were collected from the Manoel Alves River in the state of Tocantins, Brazil. Ray venom produced by venom glands was collected by scraping the epithelium of the stings, and immediately stored on ice. It was then diluted with sterile saline, homogenized, and centrifuged for collection of the supernatant, which was stored at −80 C until use. Protein content was determined using bovine serum albumin (Sigma Chemical Co., St Louis, MO) as standard. Endotoxin content was evaluated (resulting in a total dose <0.8 pg LPS) with QCL-1000 chromogenic Limulus amoebocyte lysate assay (Bio-Whittaker) according to the manufacturer’s instructions.

### Acute inflammation induced by ray venom

In order to evaluate the innate immune response induced by *Potamotrygon cf. henlei* venom, different groups of mice (*n* = 4–7) were injected intraperitoneally (i.p.) with different concentrations of venom (3, 30, and 300 μg/ml) diluted in 500 μl of sterile saline 0.9%. Thirty minutes before venom injection, different groups of mice were pretreated with a 500 μl i.p. injection containing 5 µg of neutralizing anti-mouse ST2 (ST2/IL-1R4, AF1004 - R&D Systems), 100 μM peptidyl MyD88 inhibitors (tlrl-pimyd - IMG-2005-1, Imgenex); 0.005 μM Wortamannin (Sigma, W1628); 2 μM PD98059 (Sigma) or 0.05 μM SB203580 (Sigma). Negative control mice were injected i.p. with sterile saline, and positive control mice were pretreated with i.p. injection of sterile saline, Pepinh-Control or goat IgG and submitted to i.p. application of stingray venom alone.

### Peritoneal cell suspension collection

At time points indicated (2 and 4 h, and 1, 3, 5 and 7 d) after ray venom injection, mice were killed, and peritoneal cavity exudates were harvested with 2 × 2.5 ml of cold PBS + 10 mM EDTA for cell suspensions that were centrifuged at 1500 rpm for 10 min at 4 °C. Supernatants were stored at −20 °C; and cell pellets were resuspended in 1 ml of PBS + 0.1% BSA. The total leukocyte count was performed in a 10 squares of Malassez chamber with Turk solution (20 μl cell suspension + 180 μl Turk). For differential counts, aliquots containing 100 μl of cell suspension were applied on glass slides, subjected to centrifugation at 1000 rpm for 10 min with Cytospin, stained with kit Diff-Quick Stain Set, and analyzed in an optical microscope a 40 x objective. For differential cell counts, 300 leukocytes were classified as mononuclear cells or polymorphonuclear neutrophils and counted, based on staining and morphological characteristics, using a light microscope Axio Imager A1 (Carl Zeiss, Germany) with an AxioCam ICc1 digital camera (Carl Zeiss).

### Ray venom ear injection

Ray venom at 0.3, 3, or 30 μg/ml or sterile saline was intradermally injected into the left ear pinna of mice (insulin syringe, 29-guage, Terumo). Two hours later, ear thickness was measured with a microcaliper (Keoplin, 0–10 mm or Dial Thickness Gauge, G-1A Peakock). The difference in thickness between the left and the right pinna was calculated.

### Intravital microscopy of cremaster muscle

An independent group of mice was pretreated by intrascrotal injection with 50 μl of neutralizing anti-mouse ST2 (5 µg) and 100 μM peptide inhibitors of MyD88, 0.005 μM Wortamannin, 2 μM PD98059 or 0.05 μM SB203580. After 30 min, to evaluate the effect of ray venom in microcirculation, pretreated mice received topical application of 300 μg/ml of ray venom or sterile saline in 30 µl. Negative control mice were pretreated with intrascrotal injection of sterile saline and subjected to topic application of sterile saline. Positive control mice were pretreated with an intrascrotal injection of sterile saline, Pepinh-Control or goat IgG, and subjected to topic application of ray venom alone. Mice were anesthetized with an i.p. injection of 2% xylasine - (Calmiun®, Agener União, São Paulo, SP) and with 0.5 g/Kg of ketamine (Holliday-Scott SA, Buenos Aires, Argentina). The scrotum was opened and the cremaster muscle exteriorized. After longitudinal incision with a cautery and spreading the muscle over a cover glass, the epididymis and testis were mobilized and pinned aside, allowing full microscopic access to the cremaster muscle microcirculation. The exposed tissue was superfused with 37 °C bicarbonate-buffered saline, pH 7.4. Post-capillary venules, with a diameter of 25–40 µm were chosen and the interaction of leukocytes with the luminal surface of venular endothelium was evaluated by counting the number of rolling leukocytes every 10 min after application of an inflammatory agent for 30 min. Rolling leukocytes were defined as those moving less rapidly than erythrocytes that demonstrated a clear rolling motion. The number of adherent cells was expressed as the number per 100 μm length of venule.

### Quantification of IL-1β and KC

IL-1β and KC were measured in exudates of peritoneal cavity using a specific two-site sandwich ELISA with OpEIA Kits (BD-Pharmingen, San Diego, CA, USA). Binding of biotinylated monoclonal antibodies was detected using streptoavidin-horseradish peroxidase complex and TMB (3, 3′, 5, 5′-tetramethylbenzidine) substrate solution containing hydrogen peroxide. Detection limits were 7.8 pg/ml for IL-1β and 15.6 pg/ml for KC.

### Cytometric bead array for determination the level of cytokines

Concentrations of peritoneal exudates of IL-2, IL-4, IL-6, IL-10, IFN-γ, IL-17A, and TNF-α were determined by cytometric bead array (CBA), according to the manufacturer’s protocol (560485, BD Biosciences) with minor modification. Briefly, 50 μl samples were subjected to analysis in duplicate using the cytometric bead array kit on a FACSCalibur cytometer. Cytokine concentrations were quantified using CellQuestPro and CBA software (Becton Dickinson). The detection limits for IL-2, IL-4, IL-6, IL-10, IFN-γ, IL-17A, and for TNF-α were 0.1 pg/ml, 0.03 pg/ml, 1.4 pg/ml, 16.8 pg/ml, 0.5 pg/ml, 0.8 pg/ml, and 0.9 pg/ml, respectively.

### Immunofluorescence for detection of IL-33

Ray venom (300 μg/ml) or sterile saline in 500 μl were injected i.p. into C57BL/6 *WT* or IL-33/citrine reporter (citR) mice. After 2, 4 or 8 h mice were anesthetized with a ketamine (50 mg/ml)/xylasine (25 mg/ml) and perfused through the ascending aorta with saline followed by 4% paraformaldehyde. After perfusion, spleen, liver, kidney, lung, heart, and lymph nodes were removed and post fixed for two months. For cryosectioning, fixed organs were transferred to 40% sucrose in phosphate buffer, mounted in OCT compound (Neg-50, Richard Allan), and sectioned at 15 μm on a cryotome (Cryostat HM 505E) and processed for immunofluorescence. All of the sections were stained with DAPI (sc-300415) and fluoromount-G (00-4958, eBioscience) was added to the slides prior to mounting with cover slips. IL-33-produncing cells were imaged with an inverted fluorescence microscope Olympus BX51 objective 10x, scale bar 200 μm.

### Statistical analysis

All values were expressed as mean ± SEM. Experiments were performed two times. Parametric data were evaluated using analysis of variance, followed by the Bonferroni test for multiple comparisons. Non-parametric data were assessed using the Mann-Whitney test. Differences were considered statistically significant at *p* < 0.05. The SPSS statistical package (Release 13.0, Evaluation Version, 2004) was employed.

## Electronic supplementary material

Supplementary Information
