# Supplementary material for: Stingray venom activates IL-33 producing cardiomyocytes, but not mast cell, to promote acute neutrophil-mediated injury
Source: Sci Rep. 2017 Aug 11;7:7912. doi: 10.1038/s41598-017-08395-y (PMC5554156; doi:10.1038/s41598-017-08395-y)
Supplement: Supplementary file 1 — Supplementary Information [file 41598_2017_8395_MOESM1_ESM.pdf]

**SREP-17-04813C Initial Quality Check**

**Stingray venom activates IL-33 producing cardiomyocytes, but not mast cell, to promote acute neutrophil-mediated injury**

Janaina Cardoso dos Santos, Lidiane Zito Grund, Carla Simone Seibert, Elineide Eugênio Marques, Anderson Brito Soares, Valerie F. Quesniaux, Bernhard Ryffel, Monica Lopes-Ferreira and Carla Lima

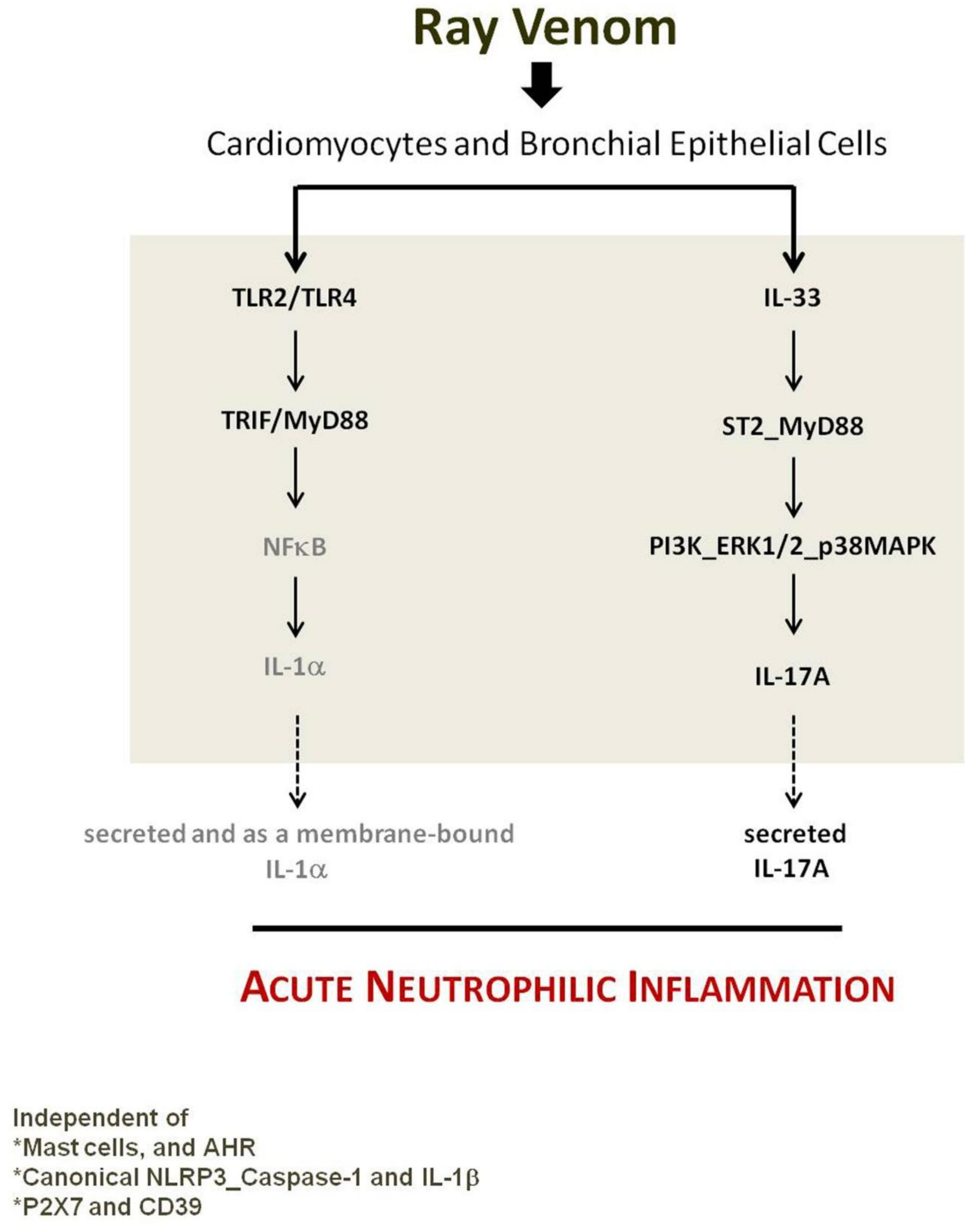

**Supplementary Figure 1. Main mechanisms involved in acute neutrophilic inflammation driven by stingray venom.** Components of the ray venom such as collagenolytic proteases can degrade extracellular matrix components, inducing epithelial cell damage accompanied by the release of IL-33 alarmin. IL-33 binds to the receptor ST2 on the cell membrane and induces recruitment of MyD88, thereby activating the downstream NF-κB, JNK, p38, and ERK pathways. Our experiments in deficient mice confirmed that ST2 engagement is critical in regulating the mobilization of neutrophils to inflamed tissue, independent of its expression on mast cells or the induction of AHR transcription factor. We found a requirement of TLR/TRIF priming signals and IL-17A for neutrophilia. IL-1β/IL-1R signaling or NLRP3_caspase-1/11 activities appeared dispensable for recruitment of neutrophils, showing that another ligand of IL-1R1 receptor as IL-1α can control the cell infiltration at the site of injury. Our data indicate that in the acute inflammation induced by ray venom, neutrophil recruitment relies on resident cell-derived IL-33, which amplifies innate immunity and cooperates to create an inflammatory microenvironment that triggers accumulation of neutrophils.
